# Vitamin D levels, general health status, and work productivity among healthcare workers—a scoping review of published literature (2010–2025)

**DOI:** 10.3389/fnut.2026.1816046

**Published:** 2026-06-02

**Authors:** Elena Vasileiou, Evangelia Nena, Paschalis Ntolios, Athanasios Zissimopoulos, Theodoros Constantinides

**Affiliations:** 1 Laboratory of Hygiene and Environmental Protection, Medical School, Democritus University of Thrace, Alexandroupolis, Greece; 2 Department of Occupational Medicine, University General Hospital of Alexandroupolis, Alexandroupolis, Greece; 3 Laboratory of Social Medicine, Medical School, Democritus University of Thrace, Alexandroupolis, Greece; 4 Department of Nursing, Faculty of Health Sciences, Democritus University of Thrace, Alexandroupolis, Greece; 5 Medical School, Democritus University of Thrace, Alexandroupolis, Greece

**Keywords:** healthcare workers, immune health, presenteeism, productivity, Vitamin D

## Abstract

**Introduction:**

The risk for Vitamin D deficiency has been reported to be elevated among healthcare workers (HCWs), a fact that raises concerns about their health, resilience, and work performance. The objective of the study is to review published literature on Vitamin D deficiency among HCWs, reported health implications as well as the association with productivity.

**Methods:**

A comprehensive search of two databases (2010–2025), PubMed and Scopus was conducted with Google Scholar used as a supplementary source following PRISMA-ScR guidelines. The inclusion criteria were full-text studies in English enrolling HCWs per WHO definition, measuring serum 25(OH)D, and reporting health or work-related outcomes.

**Results:**

Thirty-six studies met the criteria 30 cross-sectional, 2 prospective, 4 interventional studies, 2 of which were Randomized Controlled Trials (RCTs). Vitamin D deficiency was highly prevalent across studies with reported prevalence ranging from 30% to over 90% varying by geographic region, season, professional role, deficiency threshold and assay method, particularly among nurses and shift workers. Key determinants included indoor work, long shifts, night duties, limited sun exposure, female sex, younger age, higher Body Mass Index (BMI), veiling, and lack of supplementation. Seasonal variation was confirmed. Deficiency was linked to immune vulnerability (higher SARS-CoV-2 infection), musculoskeletal issues, and mixed evidence for mental health outcomes. Limited data suggest higher Vitamin D levels may reduce presenteeism. RCTs demonstrated that high-dose supplementation improved serum levels and reduced respiratory infections.

**Conclusion:**

Existing literature points that Vitamin D inadequacy among HCWs represents significant occupational health concern highlighting important evidence gaps and future research priorities.

## 1 Introduction

Healthcare workers (HCWs) constitute the backbone of all functioning health systems, worldwide. However, they often face unique challenges in the occupational setting that can compromise their physical health, mental wellbeing and their work performance (1–4). Long working hours, shift work, limited sun exposure, irregular work schedule, and high stress levels are common in healthcare settings and may contribute to a broad range of health issues, including Vitamin D deficiency (5). Vitamin D, a fat-soluble prohormone, primarily synthesized through skin exposure to solar Ultraviolet B (UVB) radiation. It plays a crucial role not only in musculoskeletal health but also in immune function, mood regulation, cardiovascular function and overall metabolic health (6–8).

Emerging evidence suggests that Vitamin D deficiency is highly prevalent among healthcare professionals, particularly those working indoors or on rotating shifts (5). Studies have shown that physicians, medical residents, nurses, and healthcare students often exhibit lower serum 25-hydroxyVitamin D [25(OH)D] levels compared to the general population, due to reduced sunlight exposure and irregular work schedules (5).

Given the ongoing debate regarding optimal serum concentrations of 25-hydroxyVitamin D, the definitions of Vitamin D deficiency, insufficiency, and sufficiency remain approximate. Multiple health authorities have converged on similar Vitamin D serum levels classifications, with slight variations in precise thresholds (9–11). National Academy of Medicine (formerly Institute of Medicine) currently defines deficiency as serum 25(OH)D levels below 12 ng/mL, insufficiency when levels are between 12 and 20 ng/mL, while sufficiency is considered when levels exceed 20 ng/mL (12). However, levels exceeding 70 ng/mL (125 nmol/L) are considered potentially toxic (13, 14) as they have been associated with adverse health outcomes, primarily due to hypercalcemia (10).

There is increasing interest in the link between micronutrient status and occupational performance (15–17). Low Vitamin D levels have been associated with fatigue, musculoskeletal pain, depressive symptoms, and diminished work efficiency (18). Among healthcare workers, these effects may translate into reduced productivity, higher absenteeism, and compromised patient care quality. Both presenteeism—where employees are physically present but unable to perform at full capacity due to health issues—and sickness absenteeism contribute to productivity losses (19). Presenteeism is typically quantified in occupational health research, using validated tools, including the Stanford Presenteeism Scale (SPS) and the Work Productivity and Activity Impairment (WPAI) questionnaire. Furthermore, given the role of calciferol in mental health and its deficiency being linked to neurodegenerative conditions and impaired cognitive function, low Vitamin D may indirectly exacerbate declines in work performance (20).

Despite a growing body of research, the precise nature of the relationship between Vitamin D status, general health, and productivity among healthcare workers remains unclear. Therefore, conducting a literature review is useful to map existing evidence, identify key concepts, and highlight knowledge gaps. This review aims to examine the association between Vitamin D levels, health outcomes, and occupational productivity among healthcare workers, with the goal of informing future research, guiding workplace wellness initiatives, and strengthening occupational health policies.

## 2 Methods

### 2.1 Protocol

This scoping review was conducted according to PRISMA- ScR guidelines (21). A formal review protocol was not preregistered in PROSPERO or OSF, as protocol registration is not consistently required for scoping reviews and PROSPERO does not universally accept scoping review protocols. Nevertheless, we used the PCC framework aligned with PICO (P- population, patient, problem, I- intervention, C- comparison, O-outcome) logic to formulate the research question which is suitable for scoping reviews: Population (P) healthcare workers (nurses, physicians, medical residents, healthcare students and allied professionals) and anyone included by WHO definition (22). Concept (C) Vitamin D levels, measured as serum 25-hydroxyVitamin D concentration, 25(OH)D deficiency and its documented or potential impact on physical health, mental health and work- related productivity outcomes (absenteeism, presenteeism, performance indicators), Context (C):in healthcare settings of any geographical region, country, climate and socioeconomic status.

### 2.2 Eligibility criteria and search criteria

To be included in this review the article could be a published study of any study design (observational, interventional, cross- sectional) that reported on healthcare workers as the primary population, measured Vitamin D status using serum 25(OH)D concentration, assessed outcomes related to general health status (e.g., fatigue, immune function, musculoskeletal health, mental health or work- related outcomes; e.g., productivity, absenteeism, presenteeism, job performance, cognitive or physical performance). The exclusion criteria were all studies without primary data, animal studies and abstract - only publications, systematic reviews and narrative reviews, population- based studies not specifically involving healthcare workers. However, secondary analyses and reviews were screened for backward citation- searching when necessary to ensure that no primary studies were missed. A meticulous search has been conducted in the following two databases: PubMed, Scopus from 1 January 2010 until 31 December 2025. Google Scholar was used exclusively as a supplementary search tool to enhance the sensitivity of the review by identifying potentially relevant studies not indexed in traditional bibliographic databases. Records were screened sequentially by relevance until retrieval saturation was reached. Accordingly, primary evidence identification and all PRISMA reporting were based solely on structured searches conducted in PubMed and Scopus.

**Figure 1:**
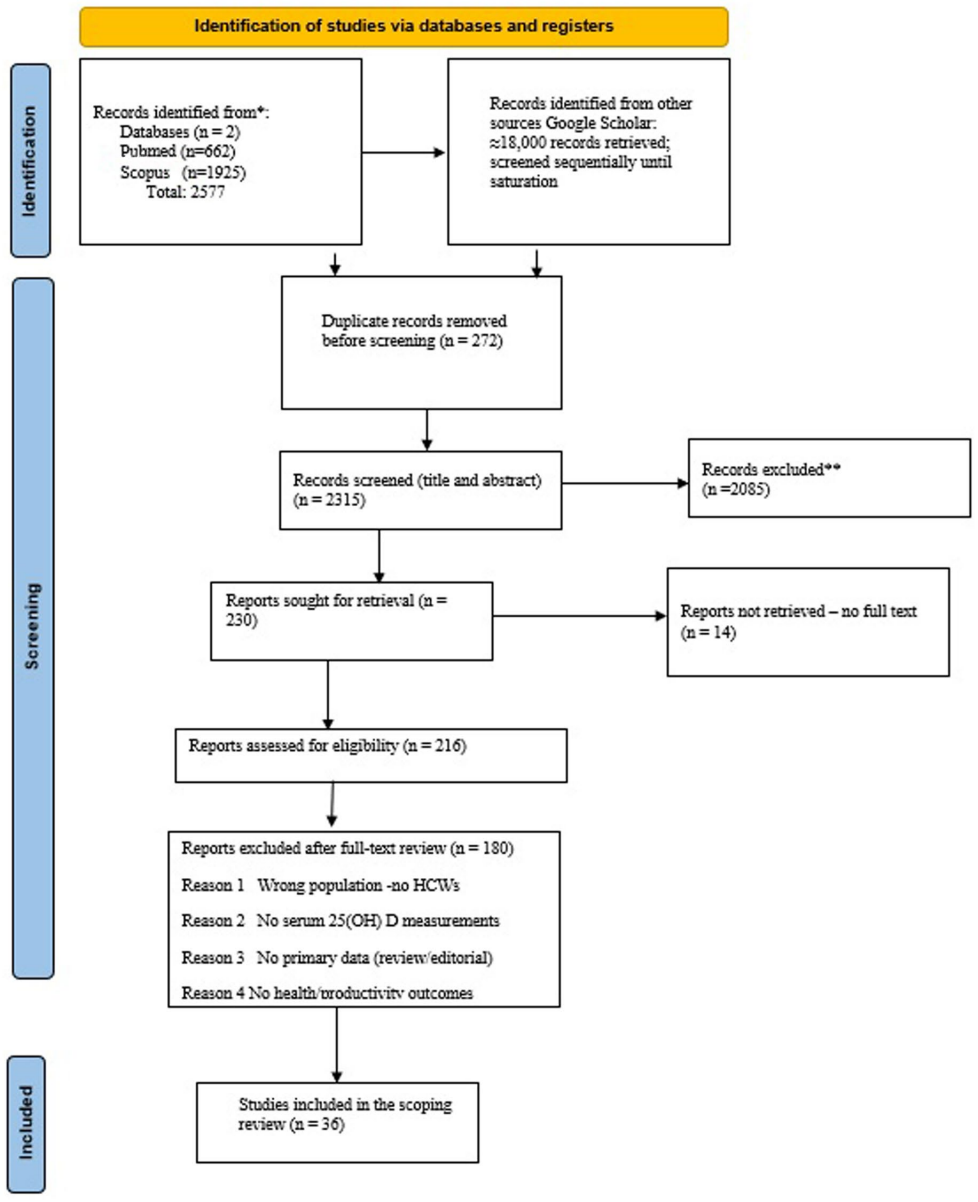
PRISMA 2020 flow diagram for the scoping review. Records were identified through systematic searches of PubMed and Scopus. Duplicate records were removed prior to screening. Google Scholar was used as a supplementary source, with records screened sequentially by relevance until saturation was reached. After title/abstract and full-text screening, 36 studies met the inclusion criteria and were included in the final scoping review. Page MJ, et al. *BMJ* 2021:372:n71. doi: 10.1136/bmj.n71. This work is licensed under CC BY 4.0.

Additional sources included reference lists of the included articles. Search strings combined PCC elements using Boolean operators were used to capture the relevant studies Supplementary material 1. The study selection process is presented in a PRISMA flow diagram (Figure 1).

All the retrieved files were imported to the reference manager (Clarivate Endnote 2025.2, Bld 19,737), duplicates were removed and two reviewers, P. N and the author E. V., independently screened title and abstracts. Any discrepancies were resolved through discussion. Data was extracted from the included studies, using a standardized charting form, capturing the following information: Author/ Year /Location/Population/Study Design/ Sample Size /Vitamin D Measurements/Outcomes Health /Productivity/Key Findings- Supplementary Tables 1–3 (Comprehensive List of the included studies). Vitamin D concentrations were standardized and reported in ng/mL across all included studies. When values were originally reported in nmol/L, systematic conversion was applied using the standard factor (1 ng/mL = 2.496 nmol/L). Original study-specific deficiency thresholds were retained and are summarized in Table 1.

**Table 1:** Deficiency thresholds and assay methods used per study.

Author (year)	Vitamin D deficiency threshold(s)	Assay method
Adeniyi et al., 2024 (23)	<20 ng/mL	High-Performance Liquid Chromatography (HPLC)
Beloyartseva et al., 2012 (24)	<20 ng/mL (deficiency); 20–29 ng/mL (insufficiency); ≥30 ng/mL (sufficiency)	Radioimmunoassay (RIA)
Chodhary et al., 2019 (25)	<20 ng/mL	Chemiluminescent Immunoassay (CLIA)
Doğruel et al., 2015 (26)	<20 ng/mL	High-Performance Liquid Chromatography (HPLC)
Funaki et al., 2022 (27)	<20 ng/mL	Chemiluminescent Enzyme Immunoassay (CLEIA)
Gannagé-Yared et al., 2014 (29)	<20 ng/mL	Chemiluminescent Immunoassay
Rajebi et al., 2016 (37)	<10 ng/mL (severe deficiency)	Electrochemiluminescent Immunoassay (ECLIA)
Haliloğlu et al., 2016 (33)	<20 ng/mL	Liquid Chromatography–Tandem Mass Spectrometry (LC–MS/MS)
Ito et al., 2024 (53)	<20 ng/mL	Electrochemiluminescent Immunoassay (ECLIA)
Jadoon et al., 2019 (31)	<20 ng/mL	Chemiluminescent Immunoassay
Lehnert et al., 2018 (35)	<20 ng/mL	Chemiluminescent Immunoassay (CLIA)
Mahdy et al., 2010 (32)	<20 ng/mL	Immunoassay
Wallingford et al., 2014 (28)	<20 ng/mL	Radioimmunoassay (RIA)
Hattapornsawan et al., 2013 (41)	<20 ng/mL	High-Performance Liquid Chromatography (HPLC)
Phelan et al., 2024 (62)	<30 ng/mL*	Immunoassay
Multani et al., 2010 (42)	<20 ng/mL	Radioimmunoassay (RIA)
Rajatanavin et al., 2019 (38)	<20 ng/mL; insufficiency 20–29 ng/mL	Liquid Chromatography–Mass Spectrometry (LC–MS)
Munter et al., 2015 (43)	<20 ng/mL; <10 ng/mL (severe)	Chemiluminescent Microparticle Immunoassay (CMIA)
Faniyi et al., 2021 (36)	<12 ng/mL†	Liquid Chromatography–Tandem Mass Spectrometry (LC–MS/MS)
Manickam et al., 2012 (39)	<30 ng/mL (insufficiency)	Immunoassay
Anuroj et al., 2022 (46)	<20 ng/mL; 20–29 ng/mL (insufficiency)	Enzyme-Linked Immunosorbent Assay (ELISA)
González-Padilla et al., 2011 (47)	<20 ng/mL	Electrochemiluminescent Immunoassay (ECLIA)
Porojnicu et al., 2012 (48)	<20 ng/mL	High-Performance Liquid Chromatography (HPLC)
Skarphedinsdottir et al., 2014 (34)	<20 ng/mL; additional thresholds at 50 and 75 nmol/L	High-Performance Liquid Chromatography (HPLC)
Mendoza et al., 2013 (40)	<20 ng/mL	Chemiluminescent Immunoassay (CLIA)
Fustinoni et al., 2025 (51)	<20 ng/mL	Liquid Chromatography–Tandem Mass Spectrometry (LC–MS/MS)
Tang et al., 2022 (44)	<20 ng/mL	Enzyme-Linked Immunosorbent Assay (ELISA)
Villasis-Keever et al., 2022 (56)	<20 ng/mL	Chemiluminescent Immunoassay
Ghammam et al., 2025 (53)	<20 ng/mL; 20–29 ng/mL (insufficiency); ≥30 ng/mL	Chemiluminescent Immunoassay (COBAS e411, Roche)
Al-Elq et al. (2012) (45)	<20 ng/mL	Chemiluminescent immunoassay (CLIA)
Van Helmond et al. (2023) (54)	<22 ng/mL	Not specified
Karonova et al. (2022) (57)	<20 ng/mL	Chemiluminescent immunoassay (CLIA)
Madani et al. (2015) (49)	Severe: <10 ng/mL; Deficiency: 10–30 ng/mL	Electrochemiluminescence immunoassay (ECLIA)
Villasis-Keever et al. (2022) (56)	Deficiency: <20 ng/mL; Insufficiency: 20–29.9 ng/mL	LC–MS/MS
Eliassen et al. (2011) (50)	Quartiles: Q1 < 18.4; Q2 18.4–<24.6; Q3 24.6–<30.6; Q4 ≥ 30.6 ng/mL (season-specific cut points also used)	RIA/LC–MS/MS (batch-adjusted
Plotnikoff et al. (2012) (55)	Severe: <10 ng/mL; Deficiency: <20 ng/mL; Insufficiency: <30 ng/mL	Chemiluminescence immunoassay

All Vitamin D concentrations are presented in ng/mL (conversion: 1 ng/mL = 2.496 nmol/L). ^*^Threshold of <30 ng/mL was used to define insufficiency/deficiency. ^†^Originally defined as <30 nmol/L; converted for consistency.

The studies have been then categorized in four thematic areas: (1) Prevalence of Vitamin D deficiency or insufficiency and associated risk factors among HCWs. (2) Health implications of Vitamin D deficiency on healthcare workers. (3) Association of Vitamin D inadequacy with work productivity. (4) The role of supplementation in addressing Vitamin D deficiency among healthcare workers.

## 3 Results

The search yielded a total of 36 publications that met the inclusion criteria and were further investigated (Figure 1). The studies were conducted between 2010 and 2025, comprising of 30 cross-sectional studies (CSSs) (Table 2), 4 interventional studies (INSs) (Table 3), two of them being Randomized Controlled Trials (RCTs) and 2 longitudinal prospective studies (Table 4).

**Table 2:** Comprehensive table of CCS studies.

Author (date)	Location/season of sampling	Aim	Population	Vit-D (assay type)	Outcomes
Adeniyi et al., 2024 (23)	Eastern Cape province of South Africa/November 2020 to February 2021.	To estimate prevalence of VDD deficiency among HCWs in association with Cardiometabolic risk factors	1,244 healthcare workers (HCWs) who completed a self-administered questionnaire and provided venous blood samples	<20 ng/mL [High-Performance Liquid Chromatography (HPLC)]	VDD prevalence 28.5% Black Africans were significantly more likely to be deficient (AOR = 2.87), as were individuals aged 42 years and older (AOR = 1.37). High rates of obesity (64.9%), hypertension (16.6%), and metabolic syndrome (18%) No significant independent association between deficiency and specific cardiometabolic markers
Beloyartseva et al., 2012 (24)	India (18 cities)/December 2010 to March 2011	To determine the prevalence of Vitamin D deficiency among healthcare professionals across different regions of India.	2,119 medical and paramedical personnel from 18 Indian cities	Deficiency: < 20 ng/mL (< 50 nmol/L) Insufficiency: 20–30 ng/mL Sufficiency: > 30 ng/mL (Radioimmunoassay)	79% were Vitamin D deficient 15% were insufficient Only 6% were sufficient Mean 25(OH)D concentration: 14.35 ± 10.62 ng/mL (median 11.93 ng/mL) No significant difference in Vitamin D levels between: Southern India: 13.3 ± 6.4 ng/mL Northern India: 14.4 ± 8.5 ng/mL
Chodhary et al., 2019 (25)	North India/sampling across multiple years from May 2017 to May 2019, covering all seasons.	To investigate the prevalence of Vitamin D deficiency among healthcare professionals, specifically doctors working in a tertiary care hospital and identify risk factors associated with low levels, such as sun exposure, diet and skin type	200 healthy doctors Working across various departments at Government Medical College & Associated Hospitals, Jammu	<20 ng/mL [Immunoassay (CLIA)]	Vitamin D deficiency prevalence: 97% (196 out of 200 doctors) Vitamin D sufficiency: Only 3% Among those deficient (n = 196): Severe deficiency: 30.41% (59/194) Moderate deficiency: 60.82% (118/194) Mild deficiency: very few Sun exposure: Majority had <20 min sun exposure per day Only 10/200 had >30 min of exposure Demographic notes: Mean age 26.18 ± 2.09 years 50% male, 50% female 65.5% Hindu, 30% Muslim
Doğruel et al. (26)	Kayseri, Turkey/November 2014–February 2015	to investigate serum 25(OH)D (Vitamin D) levels among medical staff working in different positions (dentists, dental assistants, secretaries, nurses) at the Faculty of Dentistry, Erciyes University, Turkey.	Sample: 100 staff members (51 dentists, 28 dental assistants, 11 secretaries, 10 nurses) Demographics: 62 females, 38 males; mean age 30.17 ± 5.77 years (range: 20–49) Work Schedule: All worked from 08:00 a.m. to 17:00 p.m.; mean sunlight exposure was <2 h per day for all participants. Exclusions: Those with chronic disease, drug use affecting Vitamin D metabolism, or recent Vitamin D supplementation.	<20 ng/mL [High-Performance Liquid Chromatography (HPLC)]	Vitamin D Status Mean Vitamin D level: 12.05 ± 7.42 ng/mL (range: 2.1–38.3) By occupation: Dentists: 12.08 ± 7 ng/mL Dental assistants: 10.08 ± 8.05 ng/mL Secretaries: 14.46 ± 8.62 ng/mL Nurses: 8.96 ± 5.58 ng/mL By sex: Males 13.5 ± 8.1 ng/mL; Females 11.1 ± 6.8 ng/mL (no significant difference) Prevalence: Severe deficiency (<10 ng/mL): 51 participants Deficiency (10–20 ng/mL): 36 participants Insufficiency (20–30 ng/mL): 10 participants Normal (>30 ng/mL): 3 participants (only one dentist) No significant difference in Vitamin D levels by age, sex, or occupation. No significant correlation between Vitamin D and PTH levels.
Funaki et al., 2022 (27)	Tokyo, Japan/March 1 to March 5 2021	To evaluate Vitamin D (25(OH)D), Zinc, and Natural Killer (NK) cell activity in healthcare workers (HCWs) during the COVID-19 pandemic state-of-emergency.	361 Healthcare Workers (274 women, 87 men). Median age: 35 years (range 22–67).	<20 ng/mL [Chemiluminescent Enzyme Immunoassay (CLEIA)]	COVID-related deficiency 90% of participants were deficient The findings linked this high rate of deficiency to decreased sunlight exposure due to social restrictions and indoor shifts, with severe deficiencies noted in 25.3% of men and 48.2% of women.
Gannagé-Yared et al., 2014 (29)	Lebanon/July 2011 to July 2012	Measurement of serum 25-hydroxyVitamin D (25(OH)D) levels in hospital employees, aiming to assess the prevalence of Vitamin D deficiency and to identify environmental and dietary factors influencing these levels	392 318 women 74 men Included nurses, medical assistants, technicians, secretaries, medical engineers, administrative employees, etc. Doctors and medical students were excluded.	<20 ng/mL (chemiluminescent assay)	Deficiency prevalence 15.61 ± 7.91 ng/mL, Fish consumption (*p* = 0.007) and season (*p* = 0.0001) were the only independent predictors of Vitamin D levels in a linear regression analysis. Other Factors: Levels were significantly associated with educational level (*p* = 0.03) and weekly sun exposure (*p* = 0.032) no significant correlation with BMI, age, gender, or sunscreen use
Rajebi H et al., 2016 (37)	Israel/Summer	To determine the prevalence of Vitamin D deficiency among female nurses working at Children’s Medical Center Hospital in Tehran, Iran, due to the risk factor of being a notably long period indoors and the fact that their health status may have consequences on the process of patients’ treatment	114 female nurses who were at least 20 years old entered the study voluntarily	< 10 ng/mL* [Electrochemiluminescence immunoassay (ECLIA)]	93.9% inadequate Vitamin D levels, with 69.3% classified as mean serum level was 11.7 ± 9.3 ng/mL, with the highest prevalence deficient (<10 ng/mL). The study identified that the of deficiency observed in the 26–35 age group.
Haliloğlu et al., 2016 (33)	Instabul/end of summer (peak UV exposure) and the end of winter (lowest UV exposure) to capture the full seasonal variation.	To determine seasonal Vitamin D status and its relationship with early atherosclerotic markers, endothelial function, and carotid intima-media thickness (CIMT) in healthcare workers of Marmara University Hospital, İstanbul	Healthy volunteer healthcare workers and 66 nonmedical volunteers of Marmara University Hospital were included in the study and 25-hydroxyVitamin D (25(OH)D), calcium, intact parathyroid hormone (PTH), endothelial function, and CIMT were measured twice during winter and summer seasons.	<20 ng/mL [Liquid Chromatography–Tandem Mass Spectrometry (LC–MS/MS)]	Among healthcare workers, 48.9% were deficient at the end of summer, which rose significantly to 71.5% in winter. Serum 25(OH)D was positively associated with FMD, suggesting that higher Vitamin D levels are linked to better blood vessel function
Ito et al., 2024 (30)	Tokyo, Japan/Early summer	Examine the prevalence and correlates of Vitamin D deficiency among healthcare workers 3 years after the start of the COVID-19 pandemic.	Examine the prevalence and correlates of Vitamin D deficiency among healthcare workers 3 years after the start of the COVID-19 pandemic.	Immunoassay Electrochemiluminescence immunoassay (ECLIA)	45.9% were found to be Vitamin D deficient (25[OH]D < 20 ng/mL) and 44.9% were insufficient (20–29 ng/mL) Risk Factors: Factors independently associated with a higher prevalence of deficiency included: Younger age and female sex. Smoking and lack of alcohol consumption. Lifestyle: Fewer hours of daytime outdoor activity (without sunscreen), lower intake of fatty fish, and no use of Vitamin D supplements. Occupational Impact: Notably, occupational factors—including shift work—were not independently associated with Vitamin D deficiency in this specific cohort.
Jadoon et al., 2019 (31)	Pakistan/January to March 2019.	to determine the status of Vitamin D deficiency among doctors and staff nurses working at Ziauddin University Hospital	118 56 doctors and 62 staff nurses	<20 ng/mL (chemiluminescence immunoassay)	**72%** of subjects were either deficient or insufficient.
Lehnert et al., 2018 (35) Germany	Germany/Winter to Spring, Autumn to summer	to assess the Vitamin D blood status and investigate factors that may influence serum Vitamin D levels—including age, body mass index, physical activity, and shift work—in female health care workers	67 female health care workers (aged 25–60 years)	<20 ng/mL [chemiluminescence immunoassay (CLIA)]	Median Vitamin D level across all participants was 20 ng/mL 22% of the subjects were found to be below the deficiency threshold Winter and Spring: lower Vitamin D levels (median 16 ng/mL). vs. Summer and Autumn: median 23 ng/mL Sampling in winter or spring was associated with a significant reduction in Vitamin D levels [Exp(*β*) = 0.77; 95% CI: 0.60–0.99] compared to summer and autumn. BMI and seasonality were more strong determinants for Vitamin D deficiency than shift work
Mahdy et al., 2010 (32)	Qatar/15th January 2007 and 15th January 2008	To investigate the prevalence of Vitamin D deficiency among health care professionals working at Hamad Medical Corporation in Doha, Qatar	340 healthy volunteers were included in this study	<20 ng/mL (Immunoassay)	The mean overall Vitamin D level was 11.7 ng/mL. It was lower in females (10.3 ng/mL) than in males (13.7 ng/mL). Ninety-seven percent of all participants had a mean level <30 ng/mL. Eighty-seven percent had a mean level of <20 ng/mL.
Wallingford et al., 2014 (28)	Ontario, Canada/2 distinct periods: End of winter- early spring Summer/autumn	Describe the prevalence of these conditions according to these definitions, seasonal variation in 25(OH)D and predictors of serum 25(OH)D concentrations among working, white women.	Female premenopausal nurses (n 83) working full-time rotating shifts	<20 ng/mL (RIA Radioimmunoassay)	UV exposure and Vitamin D Following winter/spring, 13% of nurses were found to have deficient While tanning beds increased Vitamin D, the authors noted the risks for premature skin aging and skin cancer outweigh these benefits. They recommended focusing on supplementation and fortified foods to protect bone health instead
Hattapornsawan Y. et al., 2013 (41)	Thailand/not mentioned	To determine the prevalence of Vitamin D (25(OH)D) deficiency and the factors influencing Vitamin D status in relation to serum 25-hydroxy Vitamin D, for example: daily sun-protective clothing, food and milk intake, sun exposure, sunscreen usage and bone mineral density (BMD).	217 nurses working at the Royal Irrigation Hospital	<20 ng/mL Not reported High-Performance Liquid Chromatography (HPLC). Dual-energy X-ray Absorptiometry (DEXA) for BMD	The combined prevalence of deficiency and insufficiency was 95.4%. The only risk factor found to have a significant correlation with Vitamin D deficiency in this group was sunscreen usage. Factors like age, BMI, and dietary habits did not show significant correlations in this specific study.
Phelan et al., 2024 (62). UK	United Kingdom/late January and early February 2021.	investigate serum Vitamin D levels in a UK population of front-line healthcare workers and to promote the occupational risk	639 volunteers was conducted to identify the prevalence of Vitamin D deficiency and insufficiency amongst a population of front-line health care workers in the UK.	<20 ng/mL (Immunoassay)	81.2% of UK frontline healthcare workers surveyed had Vitamin D levels below 75 nmol/L, a significantly higher deficiency rate than the general population. Risk factors for low levels included Asian ethnicity, younger age, male sex, lack of supplementation, and indoor shift work, leading researchers to classify this as an occupational risk factor.
Al Elq et al., 2012 (45)	Saudi Arabia/1 month- period: November 2009	Evaluate the prevalence of low levels of Vitamin D in healthy Saudi medical students.	95 male and 103 female students were analyzed	<20 ng/mL [immunoassay (CLIA)]	100% prevalence of low Vitamin D levels among medical students in Saudi Arabia, Female students showed significantly lower mean levels compared to males
Multani SK et al., 2010 (42)	Mumbai, India/not mentioned	To study BMD and the effect of environmental factors on it in resident doctors	214 healthy resident doctors were enrolled in the study.	<20 ng/mL [Radioimmunoassay (RIA) BMD Measurement: Bone Mineral Density (BMD) was measured at the lumbar spine (L1–L4), femoral neck, and total hip using Dual-energy X-ray absorptiometry (DXA)]	88.8% of total participants had Vitamin D deficiency High prevalence of low bone mass among young resident doctors with 59.7% of males and 67.5% of females exhibiting osteopenia. A significant positive correlation was found between serum 25(OH)D levels and BMD at the femoral neck, emphasizing the impact of Vitamin D on peak bone mass in young adults. Despite high ambient UV light, long indoor working hours and limited sun exposure contributed to significant Vitamin D deficiency and low bone mineral density (BMD)
Rajatanavin et al., 2019 (38)	Bangkok, Thailand/rainy season in August 2011.	Determine the prevalence of Vitamin D insufficiency among Thai dermatologists compared with the general working-age population in Bangkok and to assess sun-protective behaviors that may contribute to Vitamin D status	Dermatologists: 98 healthy Thai dermatologists (mean age 31.4 years), practicing in Bangkok, with at least 1 year of dermatology experience. Exclusions: pregnancy, breast-feeding, chronic liver/renal/bone disease, bone cancer, or taking Vitamin D/calcium supplements. General Population: 120 working-age (25–60 years) individuals residing in Bangkok, randomly selected from the Fourth Thai National Health Examination Survey (August 2008–March 2009).	<20 ng/mL 30 ng/mL [Liquid Chromatography coupled with Mass Spectrometry (LC–MS)]	Dermatologists: Mean serum 25(OH)D = 18.9 ng/mL; none had sufficient levels (>30 ng/mL); 38.8% had insufficiency (20–30 ng/mL); 61.2% had deficiency (<20 ng/mL). General population: Mean serum 25(OH)D = 26.5 ng/mL; 30.8% had sufficient levels; 50% had insufficiency; 19.1% had deficiency. Comparison: Dermatologists had significantly lower Vitamin D levels and a higher frequency of deficiency than the general population (*p* < 0.001). Sun-protective Behavior: 91% of dermatologists used sunscreen daily, spent most time indoors, and practiced various physical sun-protection activities (e.g., using a book, umbrella, long-sleeved shirt, hat).
Munter et al., 2015 (43)	Israel/winter months	to compare serum 25-hydroxyVitamin D (25(OH)D) levels among hospitalist physicians and community-based physicians, and to identify risk factors	81	<20 ng/mL [chemiluminescence microparticle immunoassay (CMIA)]	Hospital-based physicians had significantly lower mean serum 25(OH)D levels than community-based physicians (15 ± 6 vs. 19.7 ± 6 ng/mL; *p* < 0.001). Arab physicians had lower levels than Jewish physicians (11.4 ± 2.7 vs. 18.2 ± 6.6 ng/mL; p < 0.001). After excluding Arab physicians, the difference remained (hospital: 15.9 ± 6, community: 20.4 ± 6 ng/mL; *p* < 0.004). Using a cutoff of 20 ng/mL, 77% of hospital-based physicians and 68% of community-based physicians were deficient (*p* < 0.049). Using a cutoff of 10 ng/mL, 20.5% of hospital-based and 5% of community-based physicians were deficient (p < 0.049). Risk Factors: Age, night shifts, daily sun exposure, and ethnic origin were significantly linked to lower Vitamin D levels. In multivariate analysis, only ethnic origin remained significant.
Faniyi et al., 2021 (36)	United Kingdom/10-day window in May	to determine the prevalence of Vitamin D deficiency (VDD) in UK healthcare workers and to investigate the association between VDD and seroconversion for COVID-19 (i.e., evidence of previous infection)	Sample: 392 healthcare workers from University Hospitals Birmingham NHS Foundation Trust (UK), across four hospital sites. Recruitment: Staff who had isolated due to symptoms suggestive of COVID-19, recruited between 12 and 22 May 2020. Demographics: Included a range of roles (junior/senior doctors, nurses, physiotherapists, laboratory workers, etc.), with 71% White and 28% Black, Asian, and minority ethnic (BAME) participants. Exclusions: Eight participants were taking Vitamin D supplements (one in the VDD group, seven in the non-VDD group).	<12 ng/mL [liquid Chromatography-Mass Spectrometry (LC–MS/MS)]	Prevalence of Vitamin D Deficiency: 15.6% (61 out of 392) had VDD, defined as serum 25(OH)D₃ < 30 nmol/L. Risk Factors for VDD: BAME ethnicity (OR 8.86) and being a junior doctor (OR 2.15) were significant independent risk factors. Association with COVID-19 Seroconversion: Seroconversion (evidence of prior COVID-19 infection) was higher in those with VDD (72%) compared to those without (51%). VDD was an independent risk factor for seroconversion (OR 2.6). BAME males with VDD had the highest seroconversion rate (94%). Symptoms: VDD staff experienced more body aches/pains, but no significant differences in other symptoms.
Manickam et al. (39) Chicago USA 42°	Chicago, USA/Summers of 2007 and 2008	to examine determinants of serum 25-hydroxyVitamin D [25(OH)D] and bone mineral density (BMD) in young physicians—a group not well studied previously	Sample: 104 healthy medical students and residents (mean age 28.1 years, 75% women) working at an urban university hospital in Chicago, Illinois. Ethnicity: 42% White, 46% Asian, 12% “Other” (10 Hispanic, 2 African American). Exclusions: Current pregnancy, history of hyperthyroidism, liver/kidney disease, and use of medications or recreational drugs affecting bone metabolism.	<30 ng/mL Immunoassay for Vit. D Parathyroid Hormone (PTH) Bone Mineral Density (BMD) Measurement Tool: Dual-energy X-ray absorptiometry (DXA) Questionnaire: A self-administered questionnaire was used to collect data on modifiable and non-modifiable risk factors: lifestyle, environment, demographics, BMI	Vitamin D Levels: Mean serum 25(OH)D: 21.6 ng/mL (White: 27.3 ng/mL, Asian: 15.9 ng/mL, Other: 22.3 ng/mL). Vitamin D insufficiency (<30 ng/mL): 77% overall; higher in Asians (93%) than Whites (61%) and Others (73%). Vitamin D deficiency (<20 ng/mL): 48% overall; highest in Asians (67%). Determinants of Serum 25(OH)D: Age, ethnicity, exposure to sunlight, use of Vitamin D supplements, and family history of osteoporosis were significant independent determinants. Bone Mineral Density (BMD): Low BMD (osteopenia plus osteoporosis): 37.5% overall; higher in Asians (43.8%) than Whites (27.3%) and Others (50%). Significant determinants of low BMD: Sex (female protective), BMI (higher BMI protective), Asian ethnicity (borderline significance). Relationship Between Vitamin D and BMD: No significant association between serum 25(OH)D and BMD in this population. Other Findings: Serum 25(OH)D was negatively associated with serum parathyroid hormone (PTH).
Anuroj K. et al., 2022 (46)	Thailand/May 2021	to assess the prevalence of Vitamin D deficiency in Thai medical students during the COVID-19 pandemic and to determine its association with depression	Sample: 99 medical students (63 female, 36 male) in year 4 and year 5, rotating at Srinakharinwirot University Hospital, Thailand. Exclusions: Students with diseases associated with Vitamin D deficiency or who had taken Vitamin D supplements in the past year. Demographics: Age 22.8 ± 1.8 years; all Buddhist; varied family income; no pregnancy or breastfeeding reported.	<deficiency as <20 ng/mL and insufficiency as <30 ng/mL [Enzyme-Linked Immunosorbent Assay (ELISA)]	Vitamin D Levels: Mean Vitamin D level: 21.7 ng/mL. Prevalence of deficiency (<20 ng/mL): 52.5%. Prevalence of insufficiency (20–30 ng/mL): 17.2%. Adequate Vitamin D (>30 ng/mL): 30.3%. Year 4 students had significantly lower Vitamin D levels than year 5 students (12.2 vs. 30.2 ng/mL). Depression: Mean PHQ-A score: 5.8. 16 participants identified with depression (12 moderate or higher, 4 mild). Vitamin D level was not correlated with PHQ-A score or presence of depression. Only perceived difficulties score predicted depressive symptoms. Other Findings: Vitamin D level was associated with age and marginally with sex, but not with family income, GPA, perceived difficulties, psychiatric/other disease, or depressive symptoms. During the pandemic, prevalence of Vitamin D deficiency was higher than pre-pandemic Thai population estimates, likely due to reduced sunlight exposure from lockdowns and online learning.
González-Padilla E, et al., 2011 (47)	Spain/March to April	to estimate the prevalence of Vitamin D deficiency in a population of medical students of both genders from the University of Las Palmas de Gran Canaria, Canary Islands, Spain	Sample: 103 medical students (both sexes), mean age 22 years, all White, born and living in Gran Canaria. Setting: University of Las Palmas de Gran Canaria, Canary Islands, Spain	<20 ng/mL [electrochemiluminescence immunoassay (ECLIA)]	Vitamin D Status: Only 38.8% of students had 25-HCC (25(OH)D) values >30 ng/mL (optimal). 32.6% had Vitamin D deficiency (<20 ng/mL). 28.6% had Vitamin D insufficiency (20–30 ng/mL). Combined, 61.2% had either deficiency or insufficiency. Deficiency was more common in males (48.3%) than females (26.1%). Bone Health: Bone mineral density and other biochemical parameters were within normal ranges for the population. Conclusion: Despite living in a sunny climate and having knowledge about Vitamin D, nearly two-thirds of these medical students had suboptimal Vitamin D levels
Porojnicu et al., 2012 (48) Romania	Bucharest Romania/Winter – spring vs. summer - autumn	to investigate the Vitamin D status during winter in a healthy population of hospital employees in Romania and to assess the correlation between Vitamin D status and risk of upper respiratory tract infections	110 healthy hospital employees (resident doctors and nurses), mostly women (94%), mean age 35.3 years (range 20–57). Exclusions: Pregnant women were excluded. Final Analysis: 100 volunteers completed all study requirements and were included in the final analysis.	<20 ng/mL [High-Performance Liquid Chromatography (HPLC)]	Vitamin D Status:97% had serum 25(OH)D below 80 nmol/L. 80% had levels below 50 nmol/L. 35% had levels below 30 nmol/L. Only 4% had optimal levels (≥80 nmol/L). Determinants: The only significant predictor of winter Vitamin D status was sun exposure during the previous summer (*p* = 0.001). Vitamin D intake, BMI, and age were not significant predictors. Infection Risk: No statistically significant correlation was found between Vitamin D status and risk of upper respiratory tract infections during the winter (Spearman correlation coefficient = −0.12, *p* = 0.2). Other Findings: The study highlights alarmingly poor Vitamin D status in active, relatively young women in Romania, suggesting a need for public health action.
Skarphedinsdottir et al., 2014 (34)	Reykjavik, Iceland – Wisconsin, USA/Iceland: 11–18 April 2012 Wisconsin: 28 March–3 April 2011	to test whether the Vitamin D status of anesthesia department caregivers practicing at high Northern latitudes (Iceland and Wisconsin) was compatible with current recommendations, by comparing their 25-hydroxyVitamin D (25(OH)D) levels at the end of winter	106 anesthesia department caregivers in Iceland (faculty and resident physicians, non-physician anesthetists, critical care nurses) and 124 in Wisconsin (faculty and resident physicians, non-physician anesthetists). Eligibility: All participants were over 21 years old, in good health, and not taking medications affecting Vitamin D metabolism. Pregnant and lactating caregivers were excluded	<20 ng/mL [Reverse-phase high-performance liquid chromatography (HPLC)]	Mean 25(OH)D Levels: Iceland: 70.5 nmol/L (SD 30.9); Wisconsin: 70.0 nmol/L (SD 30.0); no significant difference. Prevalence: <25 nmol/L: 4.7% (Iceland), 4.0% (Wisconsin) <50 nmol/L: 34.9% (Iceland), 25.0% (Wisconsin) <75 nmol/L: 56.6% (Iceland), 61.3% (Wisconsin) Determinants: Daily multivitamin and Vitamin D supplementation were significantly associated with higher 25(OH)D levels.
Mendoza V. et al., 2013 (40).	Mexico City, Mexico/Summer 2011	to evaluate different elements of the calciotropic system (including Vitamin D, calcium, phosphorus, and parathyroid hormone) in a group of house staff physicians (medical residents), comparing them with age-, gender-, and BMI-matched controls, and to look for correlations between these elements and metabolic indices	Medical Residents: 20 healthy medical and surgical residents working at a university hospital. Controls: 20 healthy, age-, gender-, and BMI-matched non-medical individuals (young professionals working standard office hours). Exclusions: No participants were on medications affecting Vitamin D metabolism, none used sunscreen, and all had adequate dietary Vitamin D and calcium intake. Female participants were not pregnant and had regular cycles.	<20 ng/mL [chemiluminescent immunoassay (CLIA)]	Vitamin D Levels: Mean 25(OH)D was significantly lower in medical residents than controls (16.9 ± 5.1 vs. 21.5 ± 7.1 ng/mL; *p* = 0.01). Vitamin D deficiency (<20 ng/mL) was more frequent in residents (75%) than controls (45%; p < 0.001). Other Findings: Residents had significantly lower serum calcium (9.26 ± 0.3 vs. 9.55 ± 0.3 mg/dL; *p* = 0.003). PTH tended to be higher in residents, but not significantly. Vitamin D levels were inversely correlated with triglyceride concentrations (r = −0.31, *p* = 0.04). No significant differences in LDL, HDL, insulin, or HOMA-IR between groups.
Fustinoni S. et al., 2025 (51) Italy	Milan, Italy/2 distinct seasons Spring (April–May) Autumn (October–November). Monthly sampling	to evaluate the influence of night-shift work on serum and saliva levels of steroid hormones, Vitamin D, and melatonin in female hospital workers	Sample: 97 pre-menopausal female hospital workers (46 nurses performing clockwise rapid rotating shift schedule, including one night, and 51 day workers). Matching: Groups matched for age and length of service. Inclusion Criteria: Age 29–46 years, fertile status, Caucasian ethnicity, Italian language proficiency, at least 1 year of shift work for shift workers. Exclusion Criteria: Cancer, acute systemic diseases, certain medications, neurological disorders, pregnancy, obesity (BMI > 30).	<20 ng/mL [Liquid Chromatography–Tandem Mass Spectrometry (LC–MS/MS) melatonin (6-sulfatoxymelaton in urine) and a panel of steroid hormones (including cortisol, cortisone, and testosterone)]	Shift workers had: Higher levels of serum corticosterone, 11-deoxycortisol, DHEA, and androstenedione (stress-related hormones). Lower levels of estradiol and Vitamin D. No significant difference in salivary melatonin, cortisol, or cortisone circadian phase. Vitamin D: Rapid rotating shift nurses had a 13% lower concentration of Vitamin D compared to day workers (adjusted percent change: −13, 95% CI –25 to +1%). Estradiol: Lower probability of detectable estradiol in shift workers. Stress: Shift workers reported more sleep disturbance and difficulties managing family, social, and work life.
Tang et al., 2022 (44)	Taiwan/four-month period from March to June. Transition from spring to early summer	To investigate the relationship between serum 25-hydroxyVitamin D (25(OH)D) levels and mental health (depression, sleep problems, fatigue) in shift-working female nurses in Taiwan.	Sample: 95 nurses (34 dayshift, 30 evening-shift, 31 nightshift) from Kuang-Tien General Hospital, Taichung, Taiwan. Demographics: Mostly female (94–100% per group), mean age: day-shift 36.5, evening-shift 25, nightshift 25 years. Inclusion: Nurses working in fixed shifts for more than 2 months, with at least half a year of experience. Exclusion: Alcohol abuse, smoking, pregnancy/lactation, supplement use in the previous month, chronic diseases, and other health conditions	<20 ng/mL [enzyme-linked immunosorbent assay (ELISA) Chinese Health Questionnaire (CHQ-12) to assess mental health status]	Vitamin D Status Deficiency (<20 ng/mL): 48.4% of shift nurses Insufficiency (20–29 ng/mL): 43.2% Sufficiency (≥30 ng/mL): Only 8.4% Mean 25(OH)D levels: Dayshift: 21.1 ± 6.2 ng/mL; Evening-shift: 21.3 ± 6.9 ng/mL; Night-shift: 20.1 ± 4.7 ng/mL Overall: 91.6% of shift nurses had insufficient or deficient Vitamin D levels. [41598_2022.icle_18721 | PDF] Mental Health and Fatigue Fatigue: 84.2% of shift nurses experienced fatigue (average NRS-F score ≥3.3). Sleep Disturbance: Night-shift nurses had significantly more severe sleep disturbance than day- and evening-shift nurses. Depression: All groups had a high prevalence of mild depression; no significant difference in depression scores between groups. No significant correlation was found between 25(OH)D levels and depression, fatigue, or sleep disturbance when analyzed by Vitamin D status. [41598_2022.icle_18721 | PDF] Inflammatory Markers No significant differences in inflammatory markers (CRP, IL-6, TNF-*α*, IL-8, IFN-*γ*) between Vitamin D status groups
Villasis Keever et al., 2022 (52)	Mexico City, Mexico/June 2020 to January 2021.	to identify factors associated with Vitamin D (VD) deficiency in Mexican health care workers exposed to SARS-CoV-2	Sample: 468 frontline health care workers (nurses, doctors, laboratory personnel, orderlies, cleaning staff) from four tertiary care hospitals in Mexico City. Demographics: Median age 40 years; 65.4% female. Inclusion: Health care workers treating hospitalized COVID-19 patients, with blood samples for 25-hydroxyVitamin D (25[OH]D) measurement. Exclusion: Those already receiving Vitamin D or other vitamin supplements, or with unprocessed samples.	< 20 ng/mL (Chemiluminescence)	Vitamin D Status Deficiency (<20 ng/mL): 69.4% (n = 325) Insufficiency (20–29.99 ng/mL): 25.5% (n = 119) Normal (>30 ng/mL): 5.1% (n = 24) Median serum VD: 16.6 ng/mL (95% CI: 17.0–18.2) Nurses: 35.5% of sample; had the highest proportion of deficiency, but differences by role were not statistically significant. Risk Factors Type 2 Diabetes: Lower median VD (13.3 ng/mL vs. 17.1 ng/mL, *p* < 0.001); independently associated with deficiency (adjusted OR 3.40, 95% CI: 1.43–8.05). Obesity: Lower median VD (15.7 ng/mL vs. 17.1 ng/mL, *p* = 0.047); not independently significant in multivariate analysis. Comorbidities: Lower median VD (15.7 ng/mL vs. 17.6 ng/mL, p < 0.001). High Blood Pressure: No significant association. COVID-19 Infection: Higher frequency of VD deficiency among those with infection (*p* = 0.009). No significant differences by age or sex.
Ghammam et al., 2025 (53)	Sousse, Tunisia (North Africa)/October–December 2021	To determine the prevalence of chronic fatigue and CFS/ME among healthcare professionals and examine associations with sociodemographic/occupational factors, quality of life, depressive symptoms, obesity, and serum Vitamin D levels.	205 healthcare professionals at Farhat Hached University Hospital (physicians, nurses, technicians and other HCWs); administrative staff excluded.	Vitamin D3 measured using COBAS e411 analyzer (Roche kits). Vitamin D status classified as normal ≥30 ng/mL, insufficiency 21–29 ng/mL, deficiency ≤20 ng/mL	Chronic fatigue prevalence **37.1%**; CFS/ME prevalence **11.2%**. No independent association between Vitamin D levels and chronic fatigue in multivariable analysis. Obesity (adjusted OR **2.50**) and moderate-to-severe depression (adjusted OR **5.84**) increased risk; good physical (adjusted OR **0.08**) and mental QoL (adjusted OR **0.10**) were protective

**Table 3:** Comprehensive table for interventional studies.

Author (year) type	Location/season of sampling	Aim	Population	Vit-D (assay type)	Intervention (dose)	Outcomes
Van Helmond et al., (2023) (54) Pragmatic RCT	New Jersey, USA/October 2020 – November 2021	To assess whether daily Vitamin D3 supplementation at 5000 IU reduces the incidence of influenza-like illness (ILI), including COVID-19, in healthcare workers	Population: Healthcare workers aged 18 years or older at a tertiary university hospital. Sample Size: 255 healthcare workers completed at least 2 months of Vitamin D3 supplementation (intervention group); 2,827 healthcare workers served as controls. Demographics: Mean age 47 ± 12 years in the intervention group; 78% women. [nutrients-15-00180 | PDF] Exclusion Criteria: History of hypercalcemia, nephrolithiasis, intolerance to Vitamin D3, use of high-dose Vitamin D or certain medications, pregnancy, and other conditions affecting Vitamin D metabolism (see Table 2 in the paper for full list).	< 22 ng/mL Assay (not specified)	Type: Daily oral Vitamin D3 supplementation Dose: 5000 IU per day (gel capsules, Res-Q Vital D3, N3 Oceanic Inc., Pennsburg, Pennsylvania, USA) Duration: 9 months Population: Healthcare workers randomized to the intervention group, aged 18 years or older Adherence: Participants were included in the analysis if they completed at least 2 months of supplementation (to ensure adequate Vitamin D levels). Adherence was monitored via pill counts and monthly surveys	Primary Outcome: Incidence rate of all influenza-like illness (ILI) (workers referred for COVID-19 PCR testing due to ILI symptoms). Secondary Outcomes: Incidence rates of COVID-19 ILI (PCR-positive) and non-COVID-19 ILI (PCR-negative). Results: Vitamin D3 supplementation was associated with a significantly lower risk of ILI (*p* = 0.015) and non-COVID-19 ILI (*p* = 0.038). No statistically significant reduction in COVID-19 ILI (*p* = 0.152). The intervention was safe, with no serious adverse events related to Vitamin D3.
Karonova et al., (2022) (57) Randomized Controlled Trial (RCT)	Saint Petersburg, Russia/October 30, 2020, to February 28, 2021	To analyze the potential effect of Vitamin D supplementation in reducing SARS-CoV-2 infection morbidity and severity in health care workers	Population: 128 health care workers (111 women, 17 men) aged 18–65 years, working in an infectious hospital during the COVID-19 pandemic, with negative PCR for SARS-CoV-2 and no recent Vitamin D supplementation. Final Analyzed Sample: 91 randomized (38 medical doctors, 38 nurses, 15 medical attendants); after exclusions, 78 completed the study (34 doctors, 33 nurses, 11 attendants)	< 20 ng/mL (immunoassay chemiluminescent immunoassay)	Randomization: Participants were randomized 1:1 into two groups: Group I (High Dose): Water-soluble cholecalciferol 50,000 IU/week for 2 weeks, then 5,000 IU/day for the rest of the 3-month study. Group II (Standard Dose): Water-soluble cholecalciferol 2000 IU/day for 3 months. Both groups: Treatment lasted 3 months. [nutrients-.0.4-00505-v2 | PDF]	Vitamin D Status at Baseline: 60% deficient, 30% insufficient, 10% normal. Serum 25(OH)D after 3 months: Group I median 29.9 ng/mL, Group II median 26.0 ng/mL (p = 0.01); 53% of Group I and 25% of Group II reached normal Vitamin D status. SARS-CoV-2 Morbidity: No significant difference in overall infection rates between groups, but: Group I: 26% had asymptomatic SARS-CoV-2 (no clinical features). Group II: 45% had SARS-CoV-2, with half showing mild symptoms.
Madani M et al., (2015) (49) Kashan, Iran Non**-**randomized interventional (Pre–Post Observational)	Kashan, Iran/August 2013	To investigate the relationship between non-specific musculoskeletal pain and Vitamin D deficiency in female nurses	Sample: 200 female nurses, full-time, working at least 1 year in internal and surgical wards. Inclusion Criteria: Female, no chronic diseases, no recent Vitamin D supplementation, no recent fractures or musculoskeletal trauma. Exclusion Criteria: Chronic conditions (e.g., diabetes, heart disease, thyroid problems, depression, cancer), specific musculoskeletal disorders, recent Vitamin D supplementation, recent fractures or trauma	Severe deficiency: <10 ng/mL Deficiency: 10–30 ng/mL [Electro-chemiluminescence immunoassay (ECLIA)]	Standard Vitamin D correction regimen (oral supplementation)	Vitamin D Status: Mean 25-OH Vitamin D: 16.96 ng/mL 89% had Vitamin D deficiency (<30 ng/mL) 45.5% had severe deficiency (<10 ng/mL) Musculoskeletal Pain: 89% reported pain in at least one region in the previous month Most common pain: lower back (74%), knees (48.5%), upper back (41%), ankles/feet (41%) Associations: Significant relationship between Vitamin D deficiency and pain in upper/lower back and ankles/feet Vitamin D level and age explained 6.2% of the variance in total painful regions
Villasis- Keever et al., (2022) (56) Multicenter trial from four tertiary-care hospitals	Mexico City, Mexico/July 15 to December 30, 2020	To determine the efficacy and safety of Vitamin D supplementation in the prevention of SARS-CoV-2 infection in highly exposed frontline healthcare workers	321 frontline healthcare workers (physicians, nurses, laboratory technicians, radiologists, orderlies, cleaning staff). Inclusion: SARS-CoV-2 negative, not receiving Vitamin D or other vitamin supplements, no immunocompromising conditions. Randomization: 160 to Vitamin D group (VDG), 161 to placebo group (PG). 94 VDG and 98 PG completed follow-up (per-protocol analysis)	<20 ng/mL: deficiency, 20–29.9 ng/mL: insufficiency [Liquid Chromatography–Tandem Mass Spectrometry (LC–MS/MS)]	Vitamin D Group (VDG): 4,000 IU cholecalciferol (Vitamin D3) daily for 30 days (oral capsules). Placebo Group (PG): Identical-appearing placebo capsules (cornstarch) daily for 30 days. Blinding: Double-blind, parallel, placebo-controlled design. Follow-up: RT-PCR for SARS-CoV-2 at baseline and if symptoms appeared; serum 25(OH)D3 and SARS-CoV-2 IgG at baseline and day 45; adverse events monitored at days 7, 14, 21, 28, and 45	Primary Outcomes SARS-CoV-2 Infection Rate: Lower in VDG than PG (per-protocol: 6.4% vs. 24.5%, p < 0.001; ITT: 4.7% vs. 17.1%). Relative Risk (RR): 0.23 (95% CI: 0.09–0.55) per-protocol; 0.22 (95% CI: 0.08–0.59) ITT. Vitamin D Deficiency at Follow-up: Lower in VDG (per-protocol: 10.6% vs. 67.3% in PG). Secondary Outcomes Serum 25(OH)D3 Increase: Significant increase in VDG (median delta +8.8 ng/mL) vs. PG (median delta +0.59 ng/mL). Adverse Events: Similar frequency in both groups; most common were headache, constipation, and diarrhea. No serious adverse events or treatment discontinuations due to AEs. Additional Findings The protective effect of Vitamin D supplementation was independent of baseline Vitamin D status. No hospitalizations or deaths in the Vitamin D group; one severe COVID-19 case in the placebo group

**Table 4:** Comprehensive table of prospective/longitudinal studies.

Author (year)	Location/season of sampling	Aim	Population	Assay type	Outcomes
Eliassen et al., (2011) (50) USA Nested case- control set within the Nurses’ Health Study II Nested	United States/Blood samples collected between 1996 and 1999; analyses considered both overall and season-specific (summer: May–October, winter: November–April) cut points for Vitamin D	To investigate the association between plasma 25-hydroxyVitamin D (25(OH)D) levels and risk of breast cancer in predominantly premenopausal women	613 breast cancer cases and 1,218 matched controls (nested case–control study within the Nurses’ Health Study II). Eligibility: Cancer-free women aged 32–54 at blood collection, mostly premenopausal, with plasma samples collected 1996–1999. Matching: Controls matched on age, menopausal status, month/year of collection, ethnicity, luteal day, time of day, and fasting status	Q1: <18.4 ng/mL Q2: 18.4–<24.6 ng/mL Q3: 24.6–<30.6 ng/mL Q4: ≥30.6 ng/mL [Season-specific cut points also used for sensitivity analysis) (RIA/LC–MS/MS (batch-adjusted)]	No significant association between plasma 25(OH)D levels and breast cancer risk overall (top vs. bottom quartile multivariate RR = 1.20, 95% CI: 0.88–1.63, p for trend = 0.32). Subgroup Analyses: No significant association when stratified by menopausal status, age, season, or hormone receptor status. A significant positive association was observed among overweight/obese women (BMI ≥ 25), but not among lean women.
Plotnikoff et al., (2012) (55)	Minnesota and Western Wisconsin, United States/January–April 2010	to define the relationship between Vitamin D status (measured as plasma 25-hydroxyVitamin D) and employee presenteeism (reduced productivity while at work) in a large sample of health care employees	Sample: 10,646 health care employees (out of 20,692 eligible) who completed a health risk appraisal and provided a blood sample for Vitamin D measurement. Demographics: Predominantly female (87.9%), mean age 44.3 years, majority white (90.9%), various job classifications (clinical, administrative, technical, etc.)	<10 ng/mL: Severe deficiency (6.0% of sample) <20 ng/mL: Deficiency (28.9%) <30 ng/mL: Insufficiency (60.8%) (chemiluminescence immunoassay)	Mean 25-OH Vitamin D Level: 28.1 ng/mL (SD = 13.6) Presenteeism (reduced productivity at work): Employees with Vitamin D < 20 ng/mL had higher presenteeism (mean 5.58%) than those ≥20 ng/mL (mean 4.92%), *p* = 0.014. Similar significant differences for cutoffs at 30 ng/mL and 40 ng/mL. Higher Vitamin D status was associated with lower presenteeism and higher productivity. Economic Impact: Raising Vitamin D status above 20, 30, or 40 ng/mL could save 0.19, 0.55%, or 0.63% of total payroll costs, respectively—translating to $2.3–$7.7 million in potential annual savings for the employer.

A notable surge during the COVID- 19 pandemic with a clear trend consisting of pre-pandemic studies (2010–2019) mainly examined general prevalence of Vitamin D deficiency, associations with bone health, cardiometabolic risks and occupational factors like shift work and seasonal variation. Pandemic- era studies (2020 onward) show a marked increase in research on Vitamin D and immune function (COVID- 19 susceptibility, severity and seroconversion), supplementation trials aimed at preventing COVID-19 or reducing morbidity, occupational vulnerability during lockdowns and reduced sun exposure and a broader interest in Vitamin D as modifiable risk factor for infection and presenteeism. Across the board, the included studies consistently highlighted a high prevalence of Vitamin D deficiency among healthcare workers. Many also identified specific occupational risk factors contributing to this deficiency. To enhance clarity and support a comprehensive understanding of the findings, the results were systematically organized into four overarching semantic themes.

### 3.1 Prevalence of Vitamin D deficiency and insufficiency among healthcare workers and associated risk factors

A total of 25 cross-sectional studies (23–47) focused on the prevalence and risk factors of Vitamin D deficiency among healthcare workers (HCWs) across diverse settings and geographic regions. Research was conducted in Asia (India, Thailand, Pakistan, Qatar, Japan, Israel, Taiwan, Turkey, Saudi Arabia), Europe (Romania, Germany, UK, Iceland, Spain, Turkey) South Africa and America with two more recent focusing on pandemic related Vitamin D deficiency (5). All studies focused exclusively on healthcare workers (HCWs) in alignment with the definition of WHO (22), encompassing a broad spectrum of roles such as physicians, medical residents, dentists, staff nurses, medical students, as well as supportive, technical, and administrative personnel in hospital settings.

Nurses were the most frequently studied professional group with several studies reporting high prevalence of deficiency among shift- working nurses (35) and those with limited sun exposure (28, 41). Physicians and medical residents were also widely represented (39, 40, 42, 43). Two studies examined specific subgroups and medical specialties such as dental staff (26), anesthesiologists (34), dermatology personnel (38). Reported prevalence varied, ranging from approximately 30–90%, influenced by geographic location, season, and professional role. Studies from South Asia and the Middle East generally reported higher deficiency rates compared to European cohorts (24, 31, 32, 45). Seasonal variation was highlighted in Turkish healthcare workers (33), while pandemic-related studies indicated worsening deficiency during COVID-19 (27, 30). Six studies (27, 30, 31, 34, 41, 45) examined specifically sex or gender differences in Vitamin D levels among healthcare professionals. Porojnicu et al. (48) focused on female caregivers in Romania, while Lehnert et al. (35) and Wallingford et al. (28) focused on female nurses, including premenopausal staff and shift-working nurses. Tang et al. (44) studied 95 female nurses, examining associations between fatigue and sleep quality. Jadoon et al. (31) performed gender-specific analysis on 56 doctors and 62 nurses in Pakistan with women reportedly having lower Vitamin D levels due to religious veiling and less sun exposure. Risk factors for deficiency in women included young age, night shifts and irregular schedules, indoor work, poor diet, lack of supplementation, and use of protective clothing (31). Two cross-sectional studies from Thailand and Germany addressed age-related risk for Vitamin D deficiency, with contradictory findings (35, 46). Of note, in nurses, older age combined with night shifts and higher BMI increased risk (35) while among Thai medical students, younger age was associated with lower Vitamin D levels, with fourth-year students having significantly lower levels compared to fifth-year students (46). This suggests that age-related risk may vary by role and lifestyle. Four studies (33, 34, 38, 43) explicitly examined seasonal variation, with most data collected at the end of winter, a period typically associated with lower Vitamin D levels. Skarphedinsdottir et al. (34) reported seasonal deficiency among anesthesia staff, Munter et al. (43) found summer sun exposure to be the strongest predictor of Vitamin D status, Rajatanavin et al. (38) noted high insufficiency among Thai dermatologists despite the summer season. Haliloğlu et al. (33) compared summer and winter levels in Turkish healthcare workers, finding deficiency more common in winter, highlighting the need for seasonal screening and supplementation strategies. Regarding comorbidities diabetes mellitus was reported as one with a prevalence of 26% among 355 HCWs with Vitamin D deficiency (23). Higher BMI was significantly associated with lower Vitamin D levels among female night-shift nurses in Germany (35). Vascular health implications linked with metabolic conditions such as obesity were also reported in Haliloğlu et al. (33) study although this was not the primary aim of the study.

Overall, Vitamin D deficiency prevalence was not pooled into a single global estimate due to substantial heterogeneity across geographic regions, deficiency thresholds, assay methods, and occupational roles.

In South Asia, studies applying <20 ng/mL thresholds reported very high deficiency (e.g., 72–97%), whereas in Sub-Saharan Africa one large cohort reported 28.5% deficiency (<20 ng/mL). In East Asia, prevalence varied by pandemic context and season (e.g., 45.9–90% using <20 ng/mL). European cohorts showed wide variability with strong seasonal effects (e.g., 48.9% end-summer vs. 71.5% winter deficiency in Turkey). Across studies, thresholds differed (<10, <20, <30 ng/mL, and nmol/L-based cutoffs) and assays varied, limiting direct comparability. Nurses and shift workers were frequently studied, with several cohorts reporting high deficiency burdens.

### 3.2 Vitamin D deficiency and reported health implications for healthcare workers

Thirteen studies have examined how Vitamin D deficiency affects the physical and mental health of healthcare workers (HCWs) (23, 28, 33, 35, 39, 40, 42, 44, 49–53). The health outcomes investigated include immune vulnerability and susceptibility to viral respiratory infections, fatigue, muscle weakness, depression, poor bone health and non- specific musculoskeletal pain, cardiometabolic diseases, neoplasms, highlighting occupational vulnerability. Three studies examined the association of Vitamin D deficiency with cardiovascular and metabolic risks (23, 33, 49). Madani et al. (49) found that among 28% of the included HCWs had obesity, diabetes mellitus and hypertension while Haliloglu et al. (33) found that HCWs had low Vitamin D levels both in summer and winter and medical doctors reported worse endothelial function. Another study found that medical residents have low Vitamin D levels due to limited sun exposure which may affect their triglyceride levels (40). Shift work emerges as a significant determinant, with nurses and other rotating staff showing lower Vitamin D levels and related risks for bone health, skin cancer, mental wellbeing, and hormonal balance (28, 35, 44, 51). HCWs with Vitamin D deficiency showed higher rates of SARS-CoV-2 infection and symptomatic disease, suggesting a potential link between low Vitamin D and immune vulnerability (52). Multani et al. (42), examined the association of low body mass among resident doctors with Vitamin D insufficiency but no correlation was identified. While Manickam et al. (39), found that female physicians with higher BMI demonstrated lower BMI density in the lumbar region along with lower Vitamin D levels. A more recent study revealed that even rapidly rotating clockwise shift work in female healthcare workers can lead to low Vitamin D levels and is also associated with the rise of some hormones, likely associated with stress (51). In addition, although night shift nurses experienced 84.2% fatigue and sleep disturbance and had low Vitamin D levels (96.1%) depression was also present, no significant association was noted with Vitamin D levels (44). Ghammam et al. (53) assessed chronic fatigue among healthcare professionals in a North African university hospital and included serum Vitamin D measurement as a potential biological factor. While Vitamin D deficiency was common, no independent association was detected between serum 25-hydroxyVitamin D levels and chronic fatigue after adjustment, whereas obesity and depressive symptoms were significant correlates (53). Seasonal variation and pandemic-related restrictions further exacerbate deficiency, as observed during COVID-19 (27, 30). A large prospective study of US (50) found that higher circulating 25(OH)D levels may reduce breast cancer risk, particularly in premenopausal women.

### 3.3 Association between Vitamin D levels and work productivity among healthcare workers

Seven studies examined the relationship between Vitamin D status and work-related outcomes (44, 46, 52, 54–57). Plotnikoff et al. (55) conducted the largest prospective observational study to date, enrolling 10.946 HCWs. Their findings indicated a positive association between serum Vitamin D concentrations and productivity, with markedly lower rates of presenteeism among HCWs maintaining levels of Vitamin D above 40 ng/mL, with modeled estimates suggesting potential payroll savings if deficiency thresholds were addressed. However, the economic projections reported by Plotnikoff et al. (55) should be interpreted with caution as model-based estimates derived from observational associations and assumptions regarding productivity valuation, residual confounding, and generalizability. These findings are hypothesis-generating rather than causal evidence.

In addition, Anuroj et al. (46) reported high fatigue and reduced energy among deficient medical students, suggesting functional impairment and reduced academic or clinical performance. Tang et al. (44) reported high prevalence of fatigue among shift female nurses but no significant associations were established with Vitamin D deficiency.

Accordingly, Villasis Keever et al. (52) found that 69.4% of frontline healthcare workers in Mexico City were Vitamin D deficient, with obesity, diabetes, and occupational sun deprivation identified as key risk factors. This high prevalence suggested increased risks for infection-related absenteeism and reduced physical resilience. Furthermore, three interventional studies (54, 56, 57) demonstrated that high regimens of supplementations of Vitamin D effectively reduced both the incidence and the severity of respiratory viral infections. These outcomes translated into decreased healthcare utilization and fewer sick leave days among participants, underscoring the potential occupational health benefits of maintaining adequate Vitamin D levels and work ability.

### 3.4 The role of supplementation in addressing Vitamin D deficiency among healthcare workers

Six studies (26, 30, 49, 54, 56, 57) highlighted the necessity of addressing Vitamin D deficiency among health workers and the important role of Vitamin D supplementation. Two interventional studies (54, 57) demonstrated that supplementation reduced COVID-19 incidence and severity, especially in high-dose regimens (57). In addition, supplementation with Vitamin D3 5,000 IU for 9 months reduced the overall incidence of influenza-like illness (54). Ito et al. (30) and Do gruel et al. (26) supported supplementation as a preventive strategy, while Alavi et al. (49) found that Vitamin D supplementation can increase the Vitamin D levels with a notable alleviation of musculoskeletal discomfort in female nurses.

## 4 Discussion

Although individual studies exist, the literature on Vitamin D status, overall health and productivity among healthcare workers remains fragmented. The review aimed to map the existing evidence, clarify key concepts, identify research gaps, and to inform future study directions A total of 36 studies conducted over the past 15 years were included, with a notable surge during the COVID-19 pandemic (27, 30, 56, 57). Pandemic-era studies (2020 onward) focused on Vitamin D and immune function (COVID-19 susceptibility, severity, and seroconversion), supplementation trials, occupational vulnerability during lockdowns, and reduced sun exposure implicating Vitamin D as modifiable risk factor for infection and presenteeism. This surge reflects three drivers: (i) COVID-19 highlighted Vitamin D role in immune modulation, (ii) HCWs represent a high-risk group, prompting targeted studies, (iii) Increased funding and urgency for workplace health interventions.

Collectively, the evidence underscores a critical yet overlooked issue- Vitamin D deficiency among HCWs- where reported prevalence often surpasses 70%, regardless of geography or climate. Vitamin D deficiency is a global issue, affecting all age groups and regions, highly prevalent in the Middle East, South Asia and among populations with darker skin in northern latitudes (5, 58, 59). Deficiency persists even in sunny regions such as the Mediterranean, underscoring the influence of behavioral and occupational factors over solar exposure. More geographically diverse epidemiological studies are needed, along with ethnic- and sex-specific analyses to address inter- and intra-individual variability (58).

Occupational and environmental factors: indoor work, long shifts, night duties, and limited sun exposure were the most cited determinants. Additional risk factors included female sex, younger age, higher BMI, veiling, and lack of supplementation and use of sunscreen. Seasonal variation was addressed in four studies, all of which confirmed lower Vitamin D levels during winter months, reinforcing the need for seasonal screening protocols (28, 33, 34, 38).

Shift work emerged as the primary occupational determinant (28, 35, 44, 51), and this comes in alignment with the findings of another systematic review and metanalysis that shift workers demonstrate higher risk for Vitamin D hypovitaminosis compared to non- shifters (60). In addition, granted the pleiotropic role of Vitamin D (61) in human body as well the broad spectrum of occupational risks in healthcare settings, associations with other workplace exposures (e.g., chemicals) warrant further investigation.

Vitamin D deficiency was associated with a range of health concerns. Several studies linked low levels to immune dysfunction, including increased susceptibility to respiratory infections and COVID-19 (27, 36, 48, 52, 54, 56, 57). Others explored musculoskeletal health, noting associations with low bone mineral density and osteopenia particularly among young female doctors (39, 42). In addition, low vitamin levels were associated with non- specific musculoskeletal pain in nurses (49). Mental health outcomes such as fatigue, sleep disturbance, and depression were also examined, though findings were mixed (44). While some studies reported high rates of fatigue and poor sleep among deficient individuals, statistical correlations with mental health outcomes were not always significant (46).

Cardiometabolic parameters were also assessed in relation to Vitamin D, showing that medical residents had elevated indices of atherosclerosis and higher triglyceride levels (33). In addition, high prevalence of metabolic syndrome was observed among Vitamin D deficient female nurses (23).

Only a small number of studies directly assessed the impact of Vitamin D status on productivity. One large prospective study found a positive correlation between higher Vitamin D levels and productivity, particularly in reducing presenteeism (55). Other studies suggested indirect effects -such as fatigue and reduced energy- potentially impairing cognitive and physical performance, though these were rarely quantified (44). This highlights a gap in the literature and underscores the need for more targeted research on the issue.

Four studies, including two randomized controlled trials (49, 54), evaluated the role of Vitamin D supplementation among HCWs. Findings consistently indicated beneficial effects, particularly in reducing COVID-19 severity and improving immune resilience. Phelan et al. (62) advocates for recognizing Vitamin D deficiency as a modifiable occupational risk factor, urging institutional policies to include regular monitoring and supplementation programs. Accordingly, Dogruel et al. (26) recommends Vitamin D supplementation along with increase of sun exposure as preventative measures for vulnerable dental staff. While not always reducing infection rates, supplementation—especially at higher doses—may reduce symptom severity and improve immune resilience. Supplementation was well-tolerated and effective in normalizing serum levels, especially in high-dose regimens. A consensus statement further supported routine screening and supplementation in high-risk occupational groups, including HCWs (10).

Serum 25-hydroxyVitamin D concentrations above 30 ng/mL (75 nmol/L) have been associated with reduced risk for major chronic diseases such as including cardiometabolic, infectious and neurodegenerative diseases (20, 63). Currently, no supplementation is suggested for adult population, unless they belong to certain high-risk groups (10). Despite growing interest, significant gaps remain. Few studies assessed direct links between Vitamin D status and job performance, absenteeism, or cognitive functioning (55). Evidence on long-term outcomes and cost-effectiveness of supplementation programs is limited. Additionally, most research focused on nurses and physicians, neglecting other HCW categories such as technicians, administrative staff, and paramedics. Geographic and ethnic variability, as well as sex-specific differences, require further investigation to inform tailored interventions. The key domains that need further exploration on healthcare workers are those in terms with immune system modulation and the vulnerability for infections, mental health and fatigue as well as comorbidities undermining the work functioning and productivity. Although studies suggest a link between low Vitamin D and mood disorders (18), neurodegenerative diseases as well sleep disorders there is little attention to the occupational groups (20). The review supports a highly promising field of research: the broader role of micronutrients in promoting wellbeing and productivity of healthcare workers (15, 16, 64).

This review offers valuable insights but is subject to notable limitations. First, the absence of prospective protocol preregistration represents a methodological limitation. However, the review followed a predefined PCC framework and PRISMA-ScR guidance, and its methodology was established *a priori* and applied consistently throughout.

Since most studies employed a cross-sectional design, causal relationships cannot be established. Sample sizes varied widely, and demographic or methodological data were often incomplete. Few studies included quality assessments, and definitions of deficiency or measurement techniques lacked standardization. Interpretation of Vitamin D status across included studies was influenced by variability in the thresholds used to define deficiency. In the present review, the <12 ng/mL cut-off referenced in the Introduction reflects the National Academy of Medicine (NAM) definition of Vitamin D deficiency, which is primarily based on skeletal health outcomes. In contrast, most included studies applied a < 20 ng/mL threshold, consistent with thresholds commonly used in epidemiological research and clinical practice. This variability mirrors the lack of universal consensus regarding optimal 25-hydroxyVitamin D cut-offs, with definitions differing across scientific bodies, research contexts, and regional guidelines. Such heterogeneity complicates direct comparison of prevalence estimates and outcome associations across studies and should be considered when interpreting the findings (see Supplementary Table S3). In addition, substantial heterogeneity was observed in Vitamin D assay methods across studies (liquid chromatography–tandem mass spectrometry (LC–MS/MS), chemiluminescent immunoassay (CLIA), electrochemiluminescence immunoassay (ECLIA), high-performance liquid chromatography (HPLC), and radioimmunoassay (RIA)), which may contribute to variability in reported concentrations and prevalence estimates. Differences in analytical performance and calibration further limit direct inter-study comparability.

Broader research across different specialties could uncover occupational differences and risk patterns. Nurses were the most studied group among healthcare workers (28, 31, 35, 37, 41, 44, 46, 49, 51, 52) with women overrepresented—likely reflecting the workforce composition and greater willingness for participation in health-related research (65, 66). However, some evidence suggests young males may be particularly vulnerable to deficiency (38, 46, 62). In addition, most studies focus primarily on physicians and nurses. This narrow focus fails to represent the full spectrum of HCWs, such as administrative staff, technicians, paramedics, scientific and support personnel. Aside from the general observations mentioned earlier, no further evaluation of study quality or risk of bias was conducted. This was because the primary aim was to map the breadth and the scope of the existing literature. Additionally, while the 15-year time limit was considered appropriate, this temporal limitation may have excluded important earlier developments in the field. Moreover, due to the cross-sectional design of most of the included studies, causality could not be inferred. Low Vitamin D may contribute to fatigue, musculoskeletal symptoms, and reduced work functioning, but reverse causality is plausible: individuals with poorer health, higher BMI, reduced outdoor activity, or more demanding shift patterns may both have lower Vitamin D and worse outcomes. Key confounders include BMI/adiposity, physical activity, comorbidities, smoking, dietary intake, supplementation, sun-avoidance behaviors, and seasonality.

Despite these limitations, the consistency of findings across diverse settings strengthens the overall conclusions. Given the high prevalence of Vitamin D deficiency reported across healthcare worker populations, future research should prioritize the evaluation of targeted screening strategies, including the identification of subgroups most likely to benefit from assessment and intervention. In addition, well-designed prospective studies and randomized controlled trials are needed to clarify whether Vitamin D supplementation confers measurable benefits on health-related outcomes, work performance, and productivity in this occupational group. Particular attention should be given to optimal dosing strategies, duration of supplementation, and potential modifiers such as baseline Vitamin D status, seasonality, and occupational exposures.

## 5 Conclusion

This scoping review maps the available evidence on Vitamin D status in healthcare workers and highlights consistently reported Vitamin D inadequacy across many occupational settings. However, the existent literature is heterogeneous in deficiency thresholds, assay methods, and sampling season, limiting direct comparability between studies.

Evidence gaps remain substantial, particularly with respect to productivity outcomes. Standardized measures of presenteeism and absenteeism are infrequently applied, and causal inference is constrained by the predominance of cross-sectional study designs. Accordingly, rather than issuing direct clinical or policy recommendations, this scoping review identifies key priorities for future research. These include longitudinal and implementation studies evaluating targeted workplace interventions, as well as harmonized approaches to measurement and reporting. Future research should also emphasize prospective and interventional designs that assess clinically and occupationally meaningful outcomes, while further exploring the evolving role of micronutrients in supporting the health, wellbeing, and productivity of healthcare workers.

## Data Availability

The original contributions presented in the study are included in the article/Supplementary material, further inquiries can be directed to the corresponding author.
