# Supplementary material for: Vitamin D levels, general health status, and work productivity among healthcare workers—a scoping review of published literature (2010–2025)
Source: Front Nutr. 2026 Jun 2;13:1816046. doi: 10.3389/fnut.2026.1816046 (PMC13269045; doi:10.3389/fnut.2026.1816046)
Supplement: Supplementary file 1 [file Table_1.DOCX]

**Search strings for 2 databases**

A focused PubMed search strategy was used to identify studies measuring serum 25(OH)D levels in healthcare workers and reporting health or work‑related outcomes between 2010 and 2025.

**Pubmed**

  "Vitamin D"[Mesh]

  OR "25-Hydroxyvitamin D"[Mesh]

  OR "Vitamin D"[tiab]

  OR "25-hydroxyvitamin D"[tiab]

  OR "25(OH)D"[tiab]

)

AND

(

  "Health Personnel"[Mesh]

  OR "healthcare worker*"[tiab]

  OR "health care worker*"[tiab]

  OR nurse*[tiab]

  OR physician*[tiab]

  OR "medical staff"[tiab]

  OR "health personnel"[tiab]

)

AND

(

  health[tiab]

  OR "general health"[tiab]

  OR fatigue[tiab]

  OR infection*[tiab]

  OR immunity[tiab]

  OR musculoskeletal[tiab]

  OR productivity[tiab]

  OR presenteeism[tiab]

  OR absenteeism[tiab]

  OR "work performance"[tiab]

)

AND

(

  "2010/01/01"[Date - Publication]

  : "2025/12/31"[Date - Publication]

)

AND

english[lang]

**SCOPUS**

(

  TITLE-ABS-KEY(

    "vitamin D" OR "25-hydroxyvitamin D" OR "25 hydroxyvitamin D" OR "25(OH)D"

    OR calciferol* OR cholecalciferol*

  )

)

AND

(

  TITLE-ABS-KEY(

    "healthcare worker*" OR "health care worker*" OR nurse* OR physician*

    OR "medical staff" OR "health personnel" OR "health professional*" OR "medical resident*"

  )

)

AND

(

  TITLE-ABS-KEY(

    health OR "general health" OR fatigue OR infection* OR immunity OR musculoskeletal

    OR productivity OR presenteeism OR absenteeism OR "work performance"

  )

)

AND ( PUBYEAR > 2009 AND PUBYEAR < 2026 )

AND ( LANGUAGE(english) )

AND (

  LIMIT-TO(SUBJAREA,"MEDI")

  OR LIMIT-TO(SUBJAREA,"NURS")

  OR LIMIT-TO(SUBJAREA,"HEAL")

)
